# Genetically distant American *Canine distemper virus *lineages have recently caused epizootics with somewhat different characteristics in raccoons living around a large suburban zoo in the USA

**DOI:** 10.1186/1743-422X-1-2

**Published:** 2004-09-02

**Authors:** John A Lednicky, Jean Dubach, Michael J Kinsel, Thomas P Meehan, Maurizio Bocchetta, Laura L Hungerford, Nicolene A Sarich, Kelley E Witecki, Michael D Braid, Casandra Pedrak, Christiane M Houde

**Affiliations:** 1 Department of Pathology, Loyola University Medical Center, Maywood, Illinois 60153, USA; 2 Animal Molecular Genetics, Brookfield Zoo, Brookfield, Illinois 60513, USA; 3 Zoological Pathology Program, University of Illinois at Urbana-Champaign, Loyola University Medical Center, Maywood, Illinois 60513, USA; 4 Department of Animal Health, Veterinary Services, Brookfield Zoo, Brookfield, Illinois 60513, USA; 5 Cancer Immunology Program, Cardinal Bernardin Cancer Center, Department of Pathology, Loyola University Medical Center, Maywood, Illinois 60513, USA; 6 Department of Epidemiology and Preventive Medicine, University of Maryland School of Medicine, Baltimore, Maryland 21201, USA

## Abstract

**Background:**

Mortality rates have differed during distemper outbreaks among free-ranging raccoons (*Procyon lotor*) living around a large Chicago-area zoo, and appeared higher in year 2001 than in 1998 and 2000. We hypothesized that a more lethal variant of the local *Canine distemper virus *(CDV) lineage had emerged in 2001, and sought the genetic basis that led to increased virulence. However, a more complex model surfaced during preliminary analyses of CDV genomic sequences in infected tissues and of virus isolated *in vitro *from the raccoons.

**Results:**

Phylogenetic analyses of subgenomic CDV *fusion *(*F*) -, *phosphoprotein *(*P*) -, and complete *hemagglutinin *(*H*) – gene sequences indicated that distinct American CDV lineages caused the distemper epizootics. The 1998 outbreak was caused by viruses that are likely from an old CDV lineage that includes CDV Snyder Hill and Lederle, which are CDV strains from the early 1950's. The 2000 and 2001 viruses appear to stem from the lineage of CDV A75/17, which was isolated in the mid 1970's. Only the 2001 viruses formed large syncytia in brain and/or lung tissue, and during primary isolation *in-vitro *in Vero cells, demonstrating at least one phenotypic property by which they differed from the other viruses.

**Conclusions:**

Two different American CDV lineages caused the raccoon distemper outbreaks. The 1998 viruses are genetically distant to the 2000/2001 viruses. Since CDV does not cause persistent infections, the cycling of different CDV lineages within the same locale suggests multiple reintroductions of the virus to area raccoons. Our findings establish a precedent for determining whether the perceived differences in mortality rates are actual and attributable in part to inherent differences between CDV strains arising from different CDV lineages.

## Background

*Canine distemper virus *(CDV) (family *Paramyxoviridae*, genus *Morbillivirus*) is a single-stranded (negative-sense) enveloped RNA virus that is highly contagious and transmitted predominantly by aerosols [1]. Long known to cause potentially lethal disease among members of the *Canidae*, *Mustelidae*, and *Procyonidae*, CDV has recently been detected as a cause of morbidity and mortality in large felids [2], fresh-water seals (*Phoca sibirica*) [3], and various other animals. CDV killed more than 10,000 Caspian seals (*Phoca caspica*) in year 2000 [4], and decimated an African wild dog (an endangered species) breeding pack [5], demonstrating that CDV epidemics can be catastrophic. It also killed 1/3 of the Serengeti lions (*Panthera leo*) in 1994, whereas *mortality *due to CDV had not been previously described in large felids [6]. However, CDV is not uniformly lethal in related species; unlike the situation with lions, house cats (*Felis sylvestris catus*) can be infected by CDV wherein pathogenesis is unclear [7,8].

The increased importance of emerging pathogens has been most commonly attributed to changes in interactions between species or other ecological parameters [9], though changes in the pathogens or host susceptibility could also play a role. Closely related genomic variants of a particular RNA virus can arise within a host, forming a population of viruses referred to as quasispecies [10,11]. Viral quasispeciation can generate new disease patterns and broaden host ranges [10-12]. It is possible that CDV quasispeciation may account for the increasing number of clinically typical distemper cases in dogs [including those vaccinated against CDV). This implies the emergence of CDVs with different antigenic properties from the vaccine strains [5,13-15,23].

Serological tests of various captive carnivores in 1997 indicated seroconversion to CDV occurred among 28% of large felids after they were housed in outdoor exhibits at a large zoo located near Chicago (Illinois, USA) (T. Meehan and L. Hungerford, unpublished). The animals were CDV seronegative prior to outdoor display, and had not been vaccinated against CDV. Seroconversion did not occur among large felids kept indoors. It was thus apparent that the large felids acquired CDV infections during outdoor display. Distemper epizootics occur sporadically among area raccoons (*Procyon lotor*), and free-ranging raccoons were implicated as the source of CDV to the susceptible animals of the zoo, as large numbers of raccoons from adjoining forest preserves forage on the zoo grounds. The raccoons potentially transmit CDV to zoo animals indirectly through droplet infection and perhaps also through contact infection of nasal and oropharyngeal mucosa, since they are sometimes caught and consumed by zoo carnivores. Although CDV can cause high mortality in raccoons [16,17], it can also circulate widely in a population with many survivors, as documented by seroprevalence studies [18]. This suggests not only a substantial disease reservoir, but also the possibility of CDV strains with different levels of virulence. The latter notion cannot be readily resolved by current serology approaches, especially considering that CDV is presently considered monotypic by serology. For zoos where free-ranging raccoons can regularly be found, there is concern that CDV carried by raccoons might pose a health risk to susceptible collection species for two reasons: (a) CDV is highly infectious and an acknowledged lethal pathogen of many carnivores, and (b) CDV might mutate into a variant capable of broad-spectrum lethality. Wild raccoons were previously incriminated as the source of epizootics in captive carnivores in zoological collections and conservation parks [2,19]. Also, clinically apparent CDV infections occur in some omnivores such as Japanese snow monkeys (*Macaca fuscata*) [20] and collared peccaries (*Tayassu tajacu*) [21], raising the possibility that CDV might also cause lethal epidemics among non-carnivores.

Live raccoons are trapped on zoo grounds. Those with clinical neurologic signs are euthanized, necropsied, and examined for evidence of distemper or other infections. Dead raccoons found on-site are similarly evaluated whenever possible [22]. These procedures are routinely conducted as part of disease surveillance initiatives of the zoo and local and state agencies, especially because rabies is a major concern, and neurological signs that occur in distemper sometimes mimic rabies [22].

Distemper was detected in raccoons on zoo grounds in years 1998, 2000, and 2001 but not in 1999, 2002, and 2003. A total of 9/25 (36%) of the animals submitted for necropsy in 1998 and 1/14 (7%) in 2000 had lesions consistent with CDV infection. The number of animals submitted in 2001 was higher (n = 49) than for years 1998 and 2000, as was the percentage positive for CDV: 26/49 (45%). Precise data about the number of animals living within the forest preserve was not available. It was also not known whether significantly different numbers of animals utilized the zoo during the time line of this study (1998–2002). Nevertheless, there *appeared *to be a surge in distemper mortality in 2001, and comprehensive necropsy evaluations (performed by the same pathologist) revealed that the CDV lesions of the 2001 animals differed somewhat from those seen in the 1998 and 2000 animals. Since phylogenetic analyses suggest that wild-type CDVs differ according to geographical distribution rather than to host species [6,23], we asked whether a local CDV strain had mutated into a more virulent variant in 2001, causing the perceived rise in mortality and differences in histological presentation.

We first sought to identify the local lineage of CDV through direct sequence analysis of viral RNA (vRNA) in infected raccoon tissues and also attempted virus isolation from the specimens. Virus isolation was important not only to confirm direct sequence analyses but also: (a) because it was possible that direct sequence analyses might not work for various technical reasons, and (b) for future vaccine development in the event that unusual viral variants were detected for which current vaccines were ineffective. Following the example of previous investigators, we tried to obtain the identity of the circulating local CDV by determining the sequence of a subsection of the CDV *phosphoprotein *(*P*) gene, since the *P*-gene tends to remain conserved within clades of a given CDV lineage [24], and is useful for phylogenetic analysis [5,24,25]. To reduce the risk of bias arising from analysis of only one section of the CDV genome, we also examined a subsection of the CDV *fusion *(*F*) gene sequence that encodes a protein cleavage site [subtilisin-like endoprotease motif (-R-X-K/R-R-)] and the fusion domain [26]. The F-protein is the most conserved among morbilliviruses [27], and the *F*-gene sequence can be used to determine phylogenetic relationships between different morbillivirus species, such as the relationship between CDV and the closely related morbillivirus of salt-water seals called *Phocine distemper virus-1 *(PDV-1) [28]. *F-*gene analysis would thus help establish whether the virus was authentic CDV and not a related raccoon morbillivirus. Finally, the entire CDV receptorbinding *hemagglutinin (H*) gene was analyzed, since the H protein is the major determinant of tropism and cytopathogenicity [29], and is useful for phylogenetic analyses [6,23].

Whereas all the viruses were related to American CDV strains, the 1998 and 2001 viruses were clearly resolved by phylogenetic analyses into two genetically distant CDV clusters (lineages). The 2000 virus apparently stems from a sublineage related to the 2001 viruses.

## Results

### Pathology evaluation

In general, the results obtained from gross and histologic examinations of the animals were typical for CDV-induced distemper. Major findings included non-suppurative encephalitis and necrotizing bronchointerstitial pneumonia of variable severity (Table 1). As expected for wild raccoons of this area, multicentric parasitism was common, but additional underlying diseases were generally not noted. The presence of *Encephalomyocarditis virus *(EMCV) in animals 01-2641 and 01-2690, however, was unexpected.

**Table 1:** Histologic lesions of CDV-infected raccoons.

**Raccoon**	**Sex**	**M/Y^a^**	**Site^b^**	**Presentation**	**Encephalitis^f^**	**Pneumonia^h^**	**Other findings**	**EMCV^k^**
98-2645	F	8/98	FP^c^	Euthanized	++; Demyelination; axonal loss; few IB^g^	+++; Chronic; no IB	Lymphoid depletion (LN^i^); IB – other sites	-
98-2646	M	8/98	ZG^d^	Dead	-	++; Sub-acute to chronic; no IB	IB – other sites^j^	-
98-2654	M	10/98	ZG	Euthanized	Rare axonal loss	++	Ocular discharge; CDV in footpad ("Hardpad" disease); lymphoid depletion (LN and spleen)	-
98-2655	F	10/98	ZG	Dead	++; IB common	None	Lymphoid depletion (LN and spleen); IB – other sites	-
98-2666	F	12/98	ZG	Euthanized	++; Axonal loss; rare neuronal IB	++; Chronic; no IB	Lymphoid depletion (LN and spleen); IB – other sites	-
00-2601	M	1/00	ZG	Euthanized	++; Rare neuronal IB; severe axonal loss	None	IB – other sites	-
01-2641	M	5/01	OFP^e^	Euthanized	+; IB; syncytia in hippocampus	+++ with syncytia; IB	Lymphoid depletion (LN and spleen); IB – other sites	+ brain, LN, spleen)
01-2663	F	6/01	ZG	Euthanized	None	+++ with syncytia; IB	Lymphoid depletion (LN and spleen); IB – other sites	-
01-2676	F	7/01	ZG	Euthanized	+; Axonal loss; neuronal necrosis; IB; syncytia in hippocampus	+++; IB	Lymphoid depletion (LN); IB – other sites	-
01-2689	F	8/01	ZG	Euthanized	+; IB	++ with syncytia; IB	Lymphoid depletion (LN and spleen); IB – other sites; rhinitis; purulent conjunctivitis	-
01-2690	M	8/01	ZG	Euthanized	Rare neuronal necrosis; IB	None	Lymphoid depletion (LN); IB – other sites	+ (spleen)

^a^M/Y; Month and year animal examined by necropsy and specimens frozen.

^b^Site; Location where animal was trapped or found dead.

^c^FP; Forest preserve at border of zoo.

^d^ZG; Zoo grounds.

^e^OFP; Off-site forest preserve

^f^Encephalitis: -, none; +, mild; ++, moderate.

^g^IB; Characteristic intracytoplasmic or intranuclear inclusion bodies formed by *Canine distemper virus*.

^h^Pneumonia: +, mild; ++, moderate; +++, severe.

^i^LN; Lymph node.

^J^IB – other sites: Inclusion bodies in other epithelial sites.

^k^EMCV, *Encephalomyocarditis virus*.

**Figure 1:**
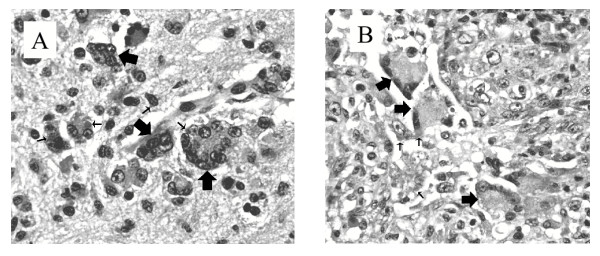
Panel A. Hematoxylin and eosin (H & E) – stained thin section of hippocampus tissue from raccoon 01-2676. Syncytia are identified by large arrows. Some CDV inclusion bodies are indicated (small arrows). Original magnification × 200. Panel B. Thin section (H & E-stained) of lung tissue from raccoon 01-2663. Syncytia and CDV inclusion bodies are identified as in panel A.

Histologic differences in the CDV lesions were apparent. While lymphoid depletion and characteristic eosinophilic intracytoplasmic inclusions in various epithelial tissues were observed in all years, inclusion bodies were more plentiful in the brain and lung tissues of raccoons examined in year 2001 than those of years 1998 and 2000. Of note, small and large (multinucleated) syncytia were present in the central nervous system and (Fig. 1A) and lung (Fig. 1B) of some raccoons from year 2001 but not in animals from 1998 and 2000 (Table 1).

### Isolation of virus from infected tissues

Virus was isolated from the tissues of 11/11 animals (Table 2) [22]. Viral cytopathic effects (CPE) in Vero cells consisted of the formation of granular-appearing cytoplasm with vacuolization (small vacuoles), followed by rounding of the cells and detachment, and *rare *formation of small stellate syncytia (consisting of 2–3 cells fused together) for viruses isolated from year 1998 and 2000 specimens or *frequent *larger rounded syncytia typically containing >8 nuclei in viruses from year 2001 [22]. Thus, the 2001 viruses appeared to form large syncytia *in vivo *(Table 1) and *in vitro *[22].

**Table 2:** CDV detection by direct RT-PCR of tissue and by virus isolation.

Raccoon	Tissue	Direct RT-PCR of Tissue	Virus isolation
98-2645	brain	-	+
98-2646	brain	-	+
98-2654	brain	+	+
98-2655	brain	-	+
98-2666	brain	+	+
00-2601	brain	+	+
01-2641	brain	+	+
	lung	+	+
	lymph node	-	+
	spleen	+	+
01-2663	brain	+	+
	lung	+	+
	lymph node	-	+
	spleen	+	+
01-2676	lung	+	+
	lymph node	+	+
01-2689	brain	+	+
	lymph node	+	+
	spleen	+	+
01-2690	brain	+	+
	kidney	-	-
	liver	-	-
	lung	+	+
	spleen	-	+

### RT-PCR and nucleotide sequence analyses

Where direct comparisons were possible, viral genomic sequence analyses indicated that the subgenomic viral *F*- and *P*- and full-genomic *H*-gene sequences did not change during *primary *isolation in three different cell lines (MDCK, MV1 Lu, and Vero [22]. Thus, for viruses from animals 98-2645, 98-2646, and 98-2655, for which direct RT-PCR from infected tissues failed (Table 2), it was likely that the sequences obtained were authentic.

**Figure 2:**
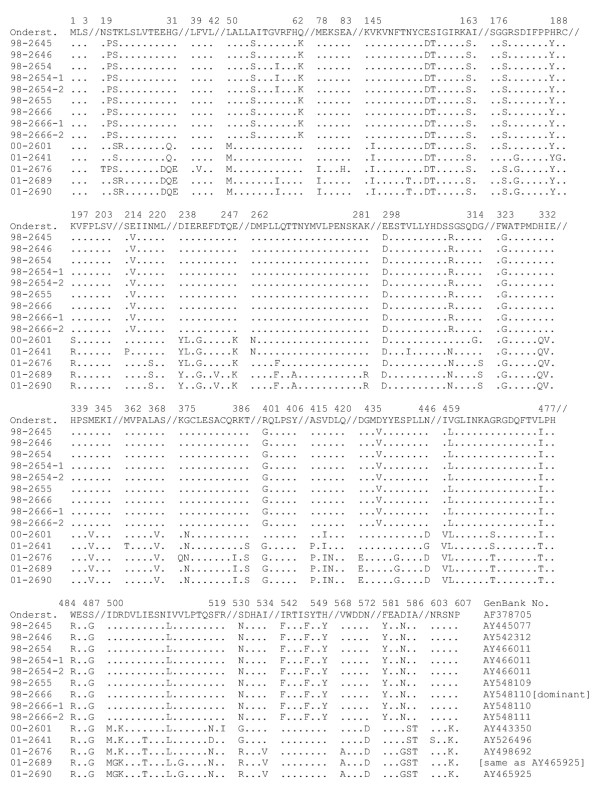
Deduced H-protein amino acid sequences of raccoon CDVs. Numbers above the sequences identify aa positions in the H-protein of CDV reference strain Onderstepoort.

**Figure 3:**
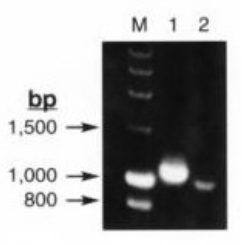
Ethidium-bromide gel electrophoresis analysis of subgenomic *H*-gene RT-PCR amplicons. For CDV-2676, shown are the 1104 bp product (lane 1) using primers CDV-HforD and CDV-Hrev75, and the 1026 bo product (lane 2) using primers CDVH-forB and CDV-HrevC (29). A 2% agarose gel was used. Molecular weight markers are loaded in the lane marked "M". Positive and negative controls were run separately and are not shown.

The subgenomic *F*- and *P- *gene of this study were previously reported [22] and deposited at GenBank (Table 3). The full-genomic *H-*gene sequences are available at GenBank (Table 3); since the *H*-gene sequences are relatively long (1,824 bp), only the deduced aa sequences are shown (Fig. 2). As for the *P*-gene, virus CDV 98-2666 had two slightly different *H*-gene sequences that were detected in vRNA in infected tissues; the same *H*-gene sequences were detected in corresponding virus isolates. The dominant *H*-gene sequence determined directly from infected tissues is labelled 98-2666 (Fig. 2, and *H*-gene sequence 98-2666 in Table 3), and is identical to the sequence of variant 98-2666-1 (Fig. 2, and *H*-gene sequence 98-2666-1 in Table 3), whereas the *H*-gene sequence of the second variant is labelled 98-2666-2. An example of RT-PCR for the CDV *H*-gene of a primary virus isolate in Vero cells is shown in figure 3.

**Table 3:** GenBank accession numbers of raccoon CDVsequences.

Virus	*F*-gene	*H*-gene	*P*-gene
98-2645	AY445077 (entire genome)		
98-2646	AY542312 (entire genome)		
98-2654-1	AY466011 (entire genome)		
98-2654-2	AY289612	(AY466011)^d^	AY286485
98-2655	(AY289612)^a^	AY548109	AY263373
98-2666-1	(AY289612)^a^	AY548110	AY286486
98-2666-2	(AY289612)^a^	AY548111	AY286487
00-2601	AY443350 (entire genome)		
01-2641-1	AY289614	AY526496	AY288310
01-2641-2	(AY289614)^b^	(AY526496)^e^	AY321298
01-2663	AY289615	ND^f^	AY288308
01-2676	(AY289615)^c^	AY498692	AY288309
01-2689	(AY289615)^c^	AY465925	AY286488
01-2690	(AY289615)^c^	(AY465925)^g^	AY264266

^a^Identical to the sequence of AY289612.

^b^Identical to the sequence of AY289614.

^c^Identical to the sequence of AY289615.

^d^Identical to the sequence of AY466011.

^e^Identical to the sequence of AY526496.

^f^ND, Not determined.

^g^Identical to the sequence of AY465925.

### Phylogenetic analyses

**Figure 4:**
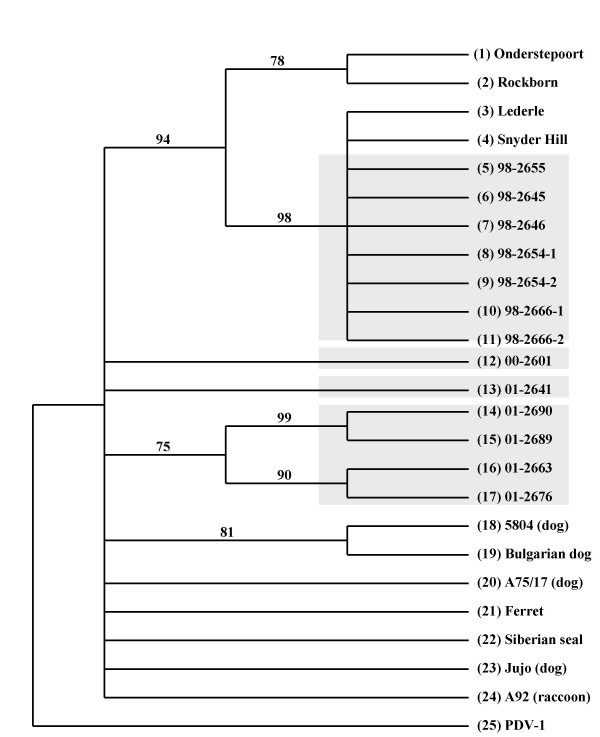
*P*-gene 70% majority rule parsimony consensus tree. Viruses from this study are high-lighted by a grey background. The animal source and GenBank numbers from top to bottom are: (1) (South African dog) AF305419, (2) (Swedish dog) AF181446, (3) (American dog) AY286480, (4) (American dog) AY286481, (5 – 17) Illinois raccoons, GenBank numbers in Table 3, (18) (German dog) AY386315, (19) (Bulgarian dog) AF259549, (20) (American dog) AF164967, (21) (German ferret) AF259550, (22) (Siberian seal) AF259551, (23) (Japanese dog) AB028916, (24) (Californa raccoon A9224/14b, reference 6), (25) (*Phocine distemper virus*) D10371.

The 70% majority-rule consensus parsimony (Fig. 4) and neighbor-joining (not shown) cladograms for the *P-*gene sequences are almost identical. Both analyses grouped the 1998 sequences together in a single clade with CDV-Lederle and -Snyder Hill with high bootstrap support. These viruses have *P*-gene sequences similar to those of CDVs Onderstepoort and Rockport, from S. Africa and Sweden, respectively. The cluster of the 2001 sequences (01-2663, -2676, -2689, -2690) was also the same in both cladograms. However, while parsimony joined the 01-2641 sequence from an offsite raccoon to the base, the distance based tree grouped this sequence with CDV A75/17. The 2000 virus was also not resolved by either method of analysis. Of the 390 bases, 34 were informative. Derivatives of the 1998 cluster form a distantly related lineage to that of 2001 cluster that is nevertheless rooted in the CDV group when compared to PDV-1 as an outgroup. CDV Lederle appears to be more derived than A9224/14b (detected in 1992 in a California (USA) raccoon [6]).

**Figure 5:**
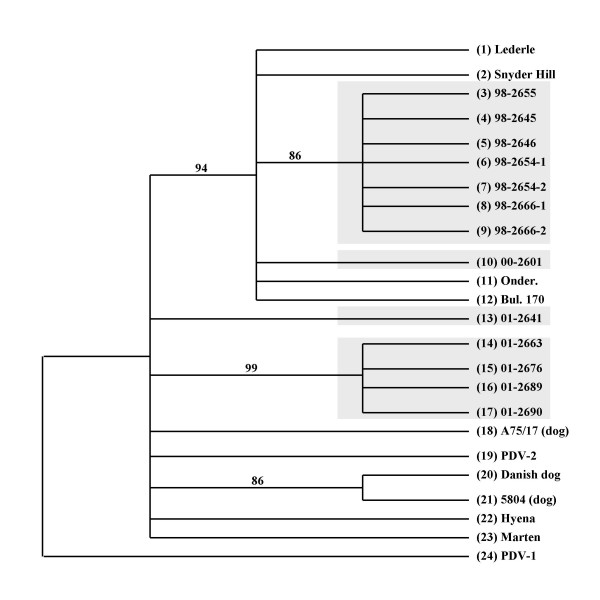
*F*-gene 70% majority rule parsimony consensus tree. Viruses from this study are high-lighted by a grey background. GenBank accession numbers are: (1) CDV Lederle (AY288311); (2) Snyder Hill (AY288312); (3 – 10, Illinois raccoons, Table 3); (11) Onder., Onderstepoort (AF378705); (12) Bul. 170, Bulgarian dog (AF259549); (13 – 17, Illinois raccoons, Table 3); (18) CDV A75/17 (AF164967); (19) PDV2, *Phocine distemper virus *2 (L07075); (20) Danish dog (AF355188); (21) CDV 5804 (from German dog) (AF026241); (22) Hyena (AF026233); (23) Marten (AF026230); (24) PDV-1 (L07075).

There were a total of 335 nucleotides in the *F*-gene and 32 of these were parsimony informative. Both parsimony (Fig. 5) and distance based (not shown) analyses produced the same topology. The off-site raccoon 01-2641 failed to group with any other sequences, joining at the base. The 1998 sequences formed a single cluster within a clade that included Lederle, Snyder Hill, and vaccine strains Onderstepoort and Bul. 170 (originally isolated from a Bulgarian dog) [30]. This clade also included the 00-2601 sequence. The remaining 2001 viruses formed a single clade with high bootstrap support.

**Figure 6:**
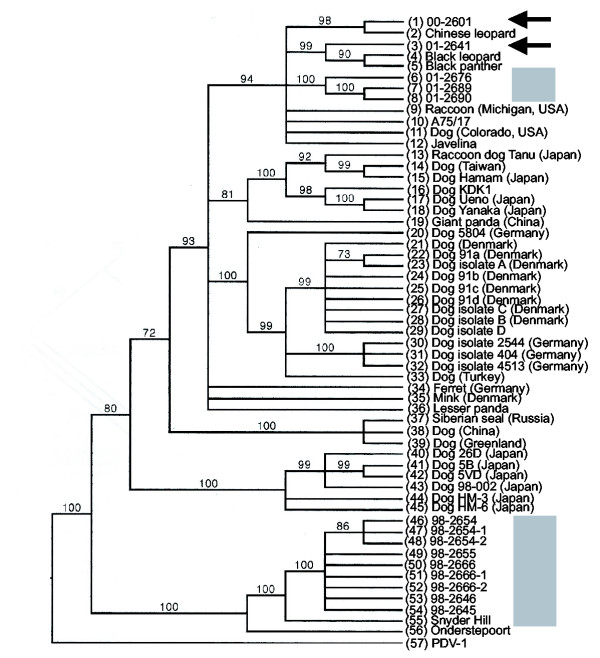
*H*-gene 70% majority rule parsimony consensus tree. Arrows or boxes demarcate locations of viruses from this study. GenBank accession numbers are: (1) CDV 00-2601 (Illinois raccoon, Table 3); (2) Chinese leopard (Z54156); (3) 01-2641 (Illinois raccoon, Table 3); (4) black leopard (Z47763); (5) black panther (Z54166); (6 – 8, Illinois raccoons, Table 3); (9) raccoon (Z47765); (10) A75/17 (AF164967); (11) dog (USA) (Z47762); (12) javelina (Z47764); (13) raccoon dog Tanu (AB016776); (14) dog (Taiwan) (AY378091); (15) dog Hamam (D85754); (16) dog KDK1 (AB025271); (17) dog Ueno (D85753); (18) dog Yanaka (D85755); (19) giant panda (AF178038); (20) dog 5804 (AY386315); (21) dog Denmark (Z47761); (22) dog 91A (AF478544); (23) dog isolate A (AF478543); (24) dog 91B (AF478546); (25) dog 91C (AF478548); (26) dog 91D (AF478550); (27) dog isolate C (AF478547); (28) dog isolate B (AF478545); (29) dog isolate D (AF478549); (30) dog isolate 2544 (Z77672); (31) dog isolate 404 (Z77671); (32) dog isolate 4513 (Z77673); (33) dog (Turkey) (AY093674); (34) ferret (X84999); (35) mink (Z47759); (36) lesser panda (AF178039); (37) Siberian seal (X84998); (38) dog (China) (AF172411); (39) dog (Greenland) (Z47760); (40) dog 26D (AB040766); (41) dog 5B (AY297453); (42) dog 5VD (AY297454); (43) dog 98-002 (AB025270); (44) dog HM-3 (AB040767); (45) dog HM-6 (AB040768); (46 – 54, Illinois raccoons, Table 3), (55) Snyder Hill (AF259552); (56) Onderstepoort (AF378705); (57) PDV-1 (AF479274).

The *H*-gene parsimony (Fig. 6) and neighbor-joining (not shown) topologies were identical with respect to the clades that include the raccoon viruses from this study. Out of 1,824 nucleotides, 420 of these were parsimony informative. As with the previous genes, the 1998 isolates and the 2000/2001 viruses formed separate clusters. The 1998 sequences joined the tree at a basal position in both analyses. The 2000 and off-site raccoon 01-2641 sequences grouped with the large felids from another zoo in Illinois.

Noteworthy, *P*-, *F*- and *H*- gene analyses indicate that the CDV sequences segregate according to geography and not to species. Since the H gene had the largest number of nucleotides, pairwise genetic distances were calculated. The 1998 isolates were most similar to the Onderstepoort and Snyder Hill (D = 4% and 1% respectively) while the 2001 isolates were most distant (D = 9% and 10% respectively). Distances within 1998 viruses were low (D ≤ 0.2%); within 2001, distances were slightly higher (D = 1%); and comparing years 1998 with 2000 and 2001, distances were highest (D = 7% to 9% respectively).

When the *P*-, *F*- and *H*- genes were combined into a single linear sequence and analyzed using parsimony and neighbor-joining algorithms with only PDV-1 as an outgroup, two independent clades are formed, the 1998 clade and the 2000/2001 clade (data not shown). In the later group, both methods join the 2000 sequence (00-2601) at a basal position to the 01-2641 off-site raccoon followed by the 2001 isolates.

## Discussion

This report shows that different CDV sublineages stemming from at least two genetically distant CDV lineages recently circulated through the local raccoon population. Our conclusion is based on numerous observations: differences in the lesions observed in animal tissues, possible dissimilarities of virulence between the viruses, variation in one viral phenotype in tissue culture (formation of large syncytia by the 2001 viruses), and from the results of nucleotide sequence and phylogenetic analyses. CDV is not maintained in hosts that recover from distemper, and persistent CDV infections do not occur. However, CDV infects a wide range of genera, and though each individual population may be small, the number of alternative host species may be substantial [1]. Forest preserves around the zoo contain many species susceptible to CDV, and it appears by inference there are separate reservoirs of different CDV lineages within the area of this study.

Since past studies indicated that wild-type CDVs differed according to geographical distribution [6,23], we initially surmised that the local CDV occasionally formed clades of highly virulent CDV variants, resulting in periodic high mortality distemper outbreaks. We also speculated that over time, highly virulent viruses would undergo extinction, and ensuing epizootics would arise from less virulent CDV variants that could affect most of the hosts without killing them. Thus, there would be an apparent oscillation (periodicity) of the mortality rates. The situation is not as straightforward, however. As shown in figures 4,5,6, at least two different CDV lineages circulated in the raccoons from 1998 – 2001. Our findings thus suggest that the outcomes of distemper might also be influenced by properties unique to different CDV lineages and their genetic variants ("strains").

The viruses from year 2001 formed syncytia *in vivo *and *in vitro*. Previously, an inverse relationship between the proficiency of syncytium formation and the level of CDV virulence was reported: the more attenuated a strain is, the higher its fusogenicity, and fusogenicity was attributed to the viral H-protein [31-34]. Therefore, the findings of this study may appear antidogmatic because increased mortality was associated with the 2001 viruses, which formed large syncytia *in vivo *and *in vitro. *However, past notions concerning the inverse relationship between fusogenicity and virulence may be imprecise. Indeed, virulent wild-type CDVs that formed syncytia in Vero cells were recently reported; the same study demonstrated that genetic changes within the *H*-gene were not required for CDV growth in Vero cells [35], as was found in this and our previous study [22]. Also, newer studies indicate that syncytium formation by CDV requires the concerted activities of both the H- and F- proteins [36-38], and that CDV virulence is the combined affect of various proteins *including *the F- and H- proteins [39]. Thus, whereas animal studies were not performed with the virus isolates of this study to directly test whether they differ in virulence, the formation of large syncytia does not rule out the possibility that the 2001 viruses are highly virulent. Noteworthy, the 2001 viruses were detected in the hippocampus and alveoli of the raccoons. Both sites were considered unusual targets of a CDV variant that was lethal to Serengeti lions, whereas CDV in dogs was said to most frequently target the brain stem and bronchi [40,41]. It is possible that tissue localization, especially with regard to the hippocampus, correlates with virus strain. In our experience, CDV in raccoons does not preferentially target the brain stem but rather infects all portions of the brain, with the possible exception of the hippocampus. We will be able to address the question whether specific CDV strains localize in the hippocampus of raccoons as we accumulate additional data from future outbreak, and after we conduct animal tests with the viruses we isolated. In contrast, CDV targets epithelial cells, and the presence of CDV in the alveoli of raccoons with distemper is common.

*H*-gene phylogenetic analyses (figure 6) suggest that a viral lineage that includes CDV A75/17 (isolated in 1975) [32] and the 2000 and 2001 viruses had infected various species including large felids [Fig. 6 and reference 6] for at least 28 years on both coasts and a midwestern state (and thus presumably throughout the continental USA). The seemingly widespread distribution suggests that viruses stemming from this lineage may be the dominant "American" CDV currently in circulation in the continental USA. The *F *-, *H*-, and *P*-gene sequence analyses (figures 4,5,6) indicate that the 1998 viruses stem from a different CDV lineage that includes American CDV strains Lederle and Snyder Hill. A recent phylogenetic analysis of the *P*-gene by an independent laboratory that utilized some of our *P*-gene data generated similar results [42]. Because they were isolated before CDV Lederle and Snyder Hill were acquired from the ATCC for this study and have distinguishable *F*- and *H*-gene sequences [22], it is certain that the 1998 CDV isolates are not due to laboratory contamination. Yet, phylogenetic analyses indicate that the CDV Lederle and Snyder Hill sequences are distant to, and in the case of the *H*-gene, ancestral to, those of the 2000 and 2001 viruses, which are as genetically distant from the 1998 viruses as they are from Snyder Hill. The source of the 1998 viruses is thus intriguing. Prior to 1997, some area raccoons were trapped, vaccinated against CDV, then released in an attempt to curtail CDV epidemics within the local raccoon population. CDV Lederle has been used as a vaccine strain in the past [3]. The vaccine used for the raccoons, (Galaxy-D, from Schering-Plough, Kenilworth, NJ), though, was made with CDV Onderstepoort, which is easily distinguished from the 1998 viruses by *F*-, *H*-, and *P*-gene analyses. However, we still could not rule out the possibility that the 1998 viruses are vaccine escape viruses from a dog vaccinated with CDV Lederle. Dogs and raccoons often frequent the same feeding sites (such as refuse disposal zones) in urban areas. The possibility of reversion to virulence of attenuated CDV exists [43], and a vaccine escape virus was proposed as a cause of distemper in a dog in Belfast, Northern Ireland [3]. We could not find a current manufacturer of anti-CDV vaccine in the USA that uses CDV Lederle. However, such vaccines were in distribution overseas around 1998 [22], and the Chicago area undergoes constant population flux, including translocation of inhabitants (and their pets) from outside of the continental USA. Related to this, the live attenuated CDV vaccine (Galaxy-D) used by the zoo up to 1997 caused vaccine-mediated distemper in different species at the zoo that had been vaccinated. For this reason, use of that particular vaccine was discontinued; instead, Purevax™, a recombinant CDV-canary pox virus vaccine (Merial, Duluth, GA) is used; the CDV insert in the canary pox virus genome is incomplete and cannot be infectious. CDV-Lederle was isolated in 1951 from a dog with encephalitis (information provided by ATCC). An alternative interpretation of our findings is that the CDV lineage that gave rise to CDV Lederle has stabilized in the local animals and is still actively circulating; more studies are needed to resolve this matter.

EMCV has been isolated or detected in raccoons before [44,45]. However, pathogenesis was uncertain, and it was thought that raccoons are a dead-end host for this virus [45]. It is known that mortality during an active case of distemper is increased in the presence of polymicrobial disease [46]. For example, a lethal outcome occurs in dogs co-infected with CDV, *Bordetella bronchiseptica*, and *Toxoplasma gondii. *It is possible that the increased mortality in 2001 was due to secondary infections with EMCV; however, no lesions attributable to EMCV were observed in pathology exams of the animals of this study, and EMCV was not isolated from all of the 2001 specimens. The significance of isolating EMCV from the brain tissue of animal 01-2641 is thus uncertain.

Our findings are especially useful for the molecular epidemiology of CDV in local wildlife, as they provide a molecular basis for CDV surveillance in area wildlife. Whereas it is considered difficult to obtain field isolates of CDV, we succeeded and can now obtain complete viral genomic sequence data (it would be difficult to do so relying solely on the limited amount of archived CDV-infected tissues from the animals of this work). Taken together, we can now monitor viral genetic drift during a long-term study of CDV in local raccoons, and will be able to conduct animal studies with the newly isolated viruses. We can also clone relevant CDV virulence genes, and express and study the biochemical properties of their specific products *in vitro*. The baseline genetic values established here will be helpful toward the development of a contemporary field-based model (since the animals are free-ranging) for studies on the emergence, evolution, maintenance, and transmission of morbilliviruses, and the efficacy of vaccines against changing viruses.

## Conclusions

The 1998 and 2001 distemper outbreaks were caused by two genetically distant American CDV lineages. Since CDV does not cause persistent infections, the cycling of different CDV lineages within the same locale suggests multiple reservoirs were responsible for the reintroduction of the virus to area raccoons. Whereas different susceptible species of the forest preserves and perhaps also some caged animals of the zoo are the most likely reservoirs, our study raises the possibility that vaccines might also be a source of CDV. The perceived differences in mortality rates that occur during intermittent distemper epizootics may be attributed in part to inherent differences between CDV strains.

## Methods

### Raccoon tissues

The raccoon tissues used in this study were described previously [22]; relevant clinical and histologic findings are presented in Table 1. Brain tissue was available for animals 98-2645, -2646, -2654, -2655, -2666 (n = 5, each collected in year 1998) and 00-2601 (n = 1, from year 2000) (Table 1). Additional tissues were available for animals 01-2641, -2663, -2676, -2689, and -2690 (n = 5, each collected in year 2001) (Table 2).

### Virus isolation

Detailed virus isolation procedures were described previously [22]. Briefly, CDV was isolated *in vitro *in MDCK, MV1-Lu, and Vero cells, eliminating the need for virus isolation in specific pathogen-free animals or in primary macrophages or other suitable cells derived thereof [29].

### RNA purification and RT-PCR

RNA purification and RT-PCR methods were previously detailed [22]. Briefly, vRNA was extracted directly from infected tissues when possible, as well as from CDV-infected tissue culture cells or from liberated CDV virions in spent cell growth media, using dedicated commercial kits (Qiagen Inc., Valencia, CA). For the American CDV strains of this work, many RT-PCR primers based on the sequence of American CDV isolate A75/17 (GenBank No. AF164967) were more effective than primers described for foreign CDV strains [22].

### Nucleic acid sequencing

Methods used for nucleic acid sequencing were previously described [22]. Briefly, all sequences were determined at least twice, starting from the purification of new RNA samples from each specimen, and both strands of each PCR amplicon were sequenced. Slab-gel sequencing utilizing dye-terminator chemistries (LI-COR, Lincoln, NE) was used at the inception of the project, then replaced by capillary sequencing using ABI-PRISM technology (Applied Biosystems, Foster City, CA). The CDV gene sequences in infected tissues were exactly like those in matched *primary *viral isolates [22]. The GenBank accession numbers for all the virus sequences of this work are given in Table 3.

### Phylogenetic analyses

Phylogenetic trees of the *P*-, *F*-, and *H*-gene sequences were constructed using the maximum-parsimony and neighbor-joining algorithms in Phylogeny Analysis Using Parsimony (PAUP) Beta Version 4.0B10 for Macintosh [47]. Heuristic searches were conducted with "simple" addition and the tree-bisection-reconnection method of branch swapping. Distance-based analyses using the minimum-evolution criterion were also conducted within PAUP using Kimura's-two-parameter model [48]. Phylogenetic tree reliability was estimated with 1000 bootstrap replications [49,50]. The appropriate *Phocine distemper virus *sequence (PDV-1) was included for outgroup rooting.

*P*-gene phylogenetic analyses were performed after an alignment of 25 *P*-gene sequences. Each *P*-gene sequence consisted of 390 ungapped positions (nucleotides 2154 to 2543 of CDV reference strain Onderstepoort) within the *P*-gene PCR amplicon. Only the internal 390 bp section of the *P*-gene PCR amplicon (432 bp) was analyzed because many relevant GenBank entries did not include the entire sequence amplified by the *P*-gene primers of this study. An additional *P*-gene sequence for raccoon A9224/14b was obtained from published data currently not deposited at GenBank [6]. Similarly, 24 ungapped *F*-gene sequences corresponding to nt 5399–5733 (335 bp) of CDV Onderstepoort were analyzed. Unlike the *P- *and *F-*genes, the entire *H*-gene was analyzed since many complete *H*-gene sequences were available at GenBank. *Phocine distemper virus 1 *(PDV1) sequences were included in the analyses for outgroup rooting.

## Competing interests

None declared.

## Authors' contributions

JAL co-conceived, designed, and coordinated the study, isolated virus, participated in the molecular genetic studies and sequence alignment, interpreted data, oversaw the training of technicians, and drafted the manuscript; JD performed phylogenetic analyses, interpreted data, and helped draft the manuscript; MJK performed pathology examinations, provided tissue specimens, helped draft the manuscript, and interpreted data; TPM co-conceived the study, provided serology data, helped draft the manuscript, and interpreted data; MB performed phylogenetic analyses, interpreted data, and helped draft the manuscript; LLH provided serology data and epidemiology perspectives, and helped draft the manuscript; NAS participated in virus isolation, molecular genetic studies, sequence alignment, and proofreading of the manuscript; KEW participated in virus isolation and molecular genetic studies, and MDB, CP, and CMH performed molecular genetic studies. All authors read and approved the final manuscript
